# Feasibility & Efficacy of Deprescribing rounds in a Singapore rehabilitative hospital- a randomised controlled trial

**DOI:** 10.1186/s12877-021-02507-0

**Published:** 2021-10-21

**Authors:** Andrew Peng Yong Wong, Tan Wan Ting, Ee Jia Ming Charissa, Tan Wee Boon, Kwan Yu Heng, Low Lian Leng

**Affiliations:** 1 Department of Post-Acute and Continuing Care, Singhealth Community Hospitals, 10 Hospital Boulevard, Singhealth Tower, Singapore, 168582 Singapore; 2 Department of Pharmacy, Singhealth Community Hospitals, 10 Hospital Boulevard, Singapore, 168582 Singapore; 3 Division of Medicine, Singapore General Hospital, Outram Road, Singapore, 169608 Singapore; 4 Program in Health Services and Systems Research, Duke-NUS Medical School, 8 College Road, Singapore, 169857 Singapore; 5 Department of Pharmacy, Faculty of Science, National University of Singapore, 21 Road, Kent Ridge, 119077 Singapore; 6 Department of Family Medicine and Continuing Care, Singapore General Hospital, Outram Road, Singapore, 169608 Singapore

**Keywords:** Deprescribing, Rounds, Multidisciplinary team, Open label, Randomised controlled trial

## Abstract

**Background:**

Deprescribing is effective and safe in reducing polypharmacy among the elderly. However, the impact of deprescribing rounds remain unclear in Asian settings. Hence, we conducted this study.

**Methods:**

An open label randomised controlled trial was conducted on patients of 65 years and above, under rehabilitation or subacute care and with prespecified medications from a Singapore rehabilitation hospital. They were randomised using a computer generated sequence.

The intervention consisted of weekly multidisciplinary team-led deprescribing rounds (using five steps of deprescribing) and usual care. The control had only usual care.

The primary outcome is the percentage change in total daily dose (TDD) from baseline upon discharge, while the secondary outcomes are the total number of medicine, total daily cost and TDD up to day 28 postdischarge, overall side-effect rates, rounding time and the challenges. Efficacy outcomes were analysed using intention-to-treat while other outcomes were analysed as per protocol.

**Results:**

260 patients were randomised and 253 were analysed after excluding dropouts (female: 57.3%; median age: 76 years). Baseline characteristics were largely similar in both groups. The intervention arm (*n* = 126) experienced a greater reduction of TDD on discharge [Median (IQR): − 19.62% (− 34.38, 0.00%) versus 0.00% (− 12.00, 6.82%); *p* < 0.001], more constipation (OR: 3.75, 95% CI:1.75–8.06, p < 0.001) and laxative re-prescriptions (OR: 2.82, 95% CI:1.30–6.12, *p* = 0.009) though death and hospitalisation rates were similar. The median rounding time was 7.09 min per patient and challenges include the inconvenience in assembling the multidisciplinary team.

**Conclusion:**

Deprescribing rounds can safely reduce TDD of medicine upon discharge compared to usual care in a Singaporean rehabilitation hospital.

**Trial registration:**

This study is first registered at Clinicaltrials.gov (protocol number: NCT03713112) on 19/10/2018 and the protocol can be accessed on https://www.clinicaltrials.gov.

**Supplementary Information:**

The online version contains supplementary material available at 10.1186/s12877-021-02507-0.

## Background

Deprescribing is the process of withdrawal of an inappropriate medication, supervised by a healthcare professional with the goal of managing polypharmacy and improving outcomes [1]. It should be perceived as part of the treatment continuum, where medications are initiated, dose-adjusted, discontinued, added or substituted, to optimise the quality of life and life expectancy [1–3].

Deprescribing has to be patient-centric with shared decisions between patients, caregivers and the healthcare team [1, 3]. Multidisciplinary interventions are generally more effective than monodisciplinary interventions in reducing inappropriate medicines [4, 5]. Five distinct steps of deprescribing had been described by Scott [2]. These include ascertaining the patient’s current medication and their indications, considering their individual risk of harm, assessing each medication’s current or future benefit, harm or burden, prioritising them for discontinuation and implementing a discontinuation regimen with close monitoring for benefits and harm.

The targets of deprescribing are myriad. We chose American Geriatric Society’s Beer’s List of Potentially Inappropriate Medicine [6] (a common guide for geriatric deprescribing) and some locally important medicine. Amongst the latter, symptomatic medicine (e.g. analgesics, laxatives) are commonly prescribed long term without clear indications [7–9]. Moreover, viscosupplements (e.g. glucosamine, chondroitin) have not demonstrated significant benefits in recent trials for the management of osteoarthritis and are potential targets for deprescribing [10, 11]. Finally, the routine supplementation of multivitamins and vitamin B complex remains controversial, beyond the replacement of individual components for established medical conditions (e.g. vitamin B12 for pernicious anaemia) [12].

Deprescribing is more critical in the elderly due to the higher prevalence of polypharmacy and inappropriate medication use [1, 13]. In Singapore, statistics from a public hospital found that more than half of all patients were discharged with at least five chronic medications [13]. Similarly, in another local study, patients in 3 nursing homes were on an average of 5.32 medications [14]. Inappropriate drug use and side effects were identified in these patients [14, 15].

Deprescribing was studied in earlier trials without causing significant adverse effects [16–19] Interventions may be initiated by either physician [16], pharmacist [19, 29] or the entire multidisciplinary team [20], using either a general [16] or specific deprescribing algorithm (e.g. palliative-geriatric methodology [17]). Positive outcomes in the elderly include reduced falls, improved quality of life and mortality [18, 21, 22]. Moreover, many deprescribing interventions had shown to reduce the total number of medicine (TNM) (up to 15%) [23] and cost (up to 20%) [17, 24]. Ward rounds are ubiquitous to inpatient care and provides an excellent opportunity for the multidisciplinary team to meet the patients. Instituting dedicated deprescribing rounds could encourage regular and consistent practice, patients’ involvement and collaboration within the doctors, nurses and pharmacists. Deprescribing rounds could safely reduce medications up to 25% [20]. However, much remains unknown about its effect in an Asian context, where healthcare perceptions and effects of deprescribing may be different from other populations [25, 26].

### Objectives

We conducted a randomised controlled trial to determine the efficacy, safety and feasibility of weekly patient-centric multidisciplinary team-led deprescribing rounds in a Singapore rehabilitation hospital.

## Methods

### Study design

We conducted an open label randomised controlled trial between 2 parallel groups (1:1) from November 2018 to August 2019. The participants were inpatients from Bright Vision Hospital, a 317 bedded Singapore rehabilitation (community) hospital. Community hospitals provide stepdown care to patients discharged from acute hospitals. Based on hospital records from 2018, approximately 78% of new admissions under rehabilitation and subacute care disciplines were more than 65 years old. Their average stay was 30 days.

All patients were screened by the study team for recruitment on the 3rd working day of admission. Ethical board approval was obtained from the centralised institutional review board. Informed consent was obtained from all participants.

### Participants

**Fig. 1:**
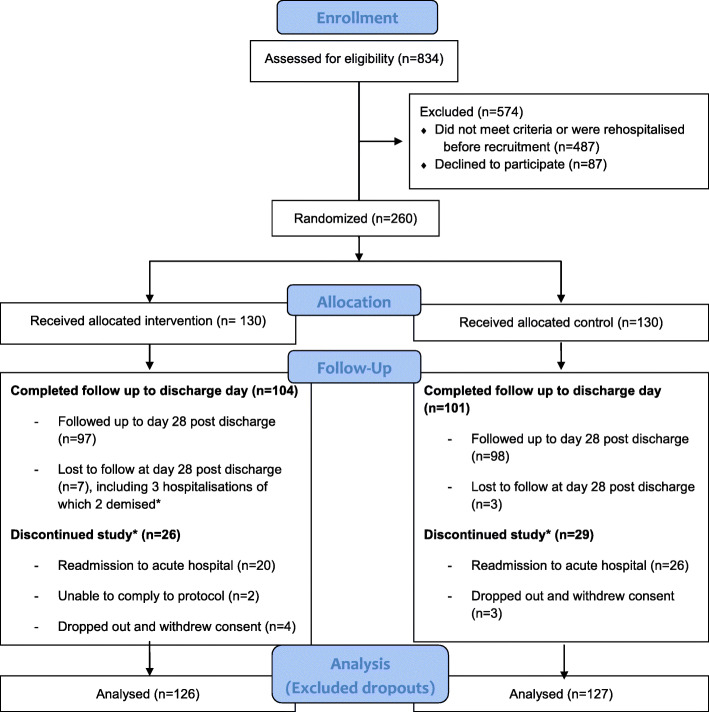
Consort flow diagram

We recruited patients who were 65 years or older, newly admitted to rehabilitation or subacute care disciplines and currently taking any of these medicines: Beer’s list of potentially inappropriate medications (American Geriatrics Society 2015 version) [6], symptomatic medications (painkillers, laxatives, antiemetics; steroid creams, gastroprotectives) and supplements (chondroitin, glucosamine, multivitamins, vitamin B complex). We excluded patients who scored below 7 on the Abbreviated Mental Test (AMT), had no mental capacity or could not provide informed consent. The study was discontinued for patients who were readmitted to acute hospitals, dropped out or were noncompliant to the protocol (Fig. 1). Crossover between groups was not permitted.

### Randomisation

Patients were randomised to either intervention or control. An independent administrator in a central office generated the entire randomisation sequence using GraphPad randomisation sequence software© 2017 (simple randomisation) before recruitment commenced. This data was password protected and only accessible by her. The individual allocation was revealed to each patient, their ward doctors, the deprescribing and study teams just before the first deprescribing round, hereby ensuring allocation concealment.

### Intervention

The intervention comprised of weekly scheduled deprescribing rounds at the patient’s bedside, conducted by a dedicated multidisciplinary team (a non-ward doctor, a central pharmacist, a ward nurse). The investigators were the non-ward doctor and the central pharmacist. During these rounds, the team applied Scott’s five steps of deprescribing [2] and checklisted against the predetermined target medicine. After deprescribing, they informed the ward doctors immediately of changes and subsequently the ward team implemented the changes. (None of the ward doctors had special training in deprescribing). The intervention group received such rounds from the day of recruitment to the day of discharge, on top of usual care.

The deprescribing criteria included the absence of clear indications or benefits of these medicine, symptom resolution, medicine side effects or risks that outweigh their benefits, and the patient’s final preference after discussing with the deprescribing team. We provided training to the multidisciplinary team on conducting the intervention and collecting outcomes with the use of standardised interview scripts, and ran pilot rounds for familarisations before the trial started.

### Control

The control group received usual care by the ward doctors, including regular ward rounds, medication changes and monitoring side effects at their own discretion.

### Outcomes

The primary outcome is an efficacy outcome which is the percentage reduction of total daily dose (TDD) of medications from baseline upon inpatient discharge. (TDD is the maximum number of doses a patient takes a day and is a marker of pill burden.) [27]

Secondary outcomes include other efficacy outcomes (namely percentage reduction of TDD at 3 other timepoints mentioned below, TNM and total daily cost of medicine [TDC] at all 4 timepoints), safety outcomes (i.e. recurring or worsening symptoms; medicine reinitiation or substitution, hospitalisation and death) and the feasibility of implementing the intervention. We adopted the threshold of the current level of symptoms at the point of deprescribing of medicine^32^. That could be either zero symptoms or minimal tolerable stable symptoms. Recurrence of symptoms was taken to be any symptom which is worse than this threshold [31]. Feasibility was measured by the rounding time taken per patient and by documenting the multidisciplinary deprescribing team’s response to this question “What were the challenges you faced in this round?”

The timepoints are days 14 and 28 of inpatient recruitment (where applicable), discharge day and day 28 postdischarge. The former 2 timepoints were chosen to explore the influence of a longer inpatient stay on efficacy outcomes. The discharge day was chosen as it marked the end of the intervention and day 28 postdischarge was chosen to explore if there are sustained effects of the intervention.

### Data collection

The deprescribing team collected all outcomes. Baseline characteristics were collected upon recruitment. This included demographic data (age, gender, ethnicity) and clinical data (the discipline upon admission, AMT scores, medical conditions, initial deprescribing targets). Each patient’s length of stay was recorded on their discharge. Efficacy outcomes (TDD, TNM, TDC) were collected on days 14 and 28 of inpatient recruitment (where applicable), discharge day and day 28 postdischarge. Safety outcomes (worsening or recurring symptoms, medicine reinitiation or substitution, rehospitalisation or death, if any) were collected weekly, on discharge and on day 28 postdischarge. Feasibility outcomes (rounding time and challenges) were collected weekly.

TDC was calculated using the selling price of medicine based on the institution’s formulary, excluding government taxes and subsidies. If this was unavailable (e.g. when patients had preexisting branded medicine), the lowest selling price in private pharmacies was used. The rounding time per patient was measured as the time taken to conduct the five steps of deprescribing, complete the deprescribing checklists and inform the ward doctors.

All outcomes were obtained using both inpatient records and interviews using standardised scripts (i.e. bedside interviews during the inpatient phase of the study; telephone interviews during outpatient phase). If the latter was not possible, clinic or home visits were offered with fees waived.

### Measures to mitigate bias

We used simple randomisation and ensured allocation concealment to prevent preferential recruitment of patients to either group.

All clinicians and patients were not blinded as they need to know the deprescribing plans for safe participation. To reduce interviewer bias for subjective outcomes, we used a standardised interview script so that leading questions are not asked. (e.g. all patients were asked specifically if the pain experienced was better, the same or worse than the preceding week when painkillers were deprescribed). To reduce reporting bias, we collected all objective data (e.g. inpatient medicine records for efficacy outcomes and medicine represcriptions, nursing records for bowel and vomiting episodes). Moreover, data was deidentified for the analyst and one ward doctor who assisted to adjudicate death and hospitalisation outcomes.

We mitigated the risk of contamination in this single study site by reporting if there were ward doctors with special training in deprescribing and planned to adjust for this in the analysis. We chose nonward doctors to be part of the multidisciplinary team so that they might not apply the intervention to the control group. We ensured their compliance to the intervention by providing them with all the checklists (5 steps of deprescribing, list of target medicine) before rounds and ensured all tasks were performed and checkboxed by the end of the rounds.

### Statistical methods

We used PS Power and Sample Size Calculations version 3.1.2. Based on Roberts’ study [23], assuming deprescribing rounds would produce a 15% reduction in TDD, 110 patients were needed in each group (α = 0.05, using a 2sided test, β = 0.2). After factoring possible study discontinuation (15%), 260 patients were required.

Efficacy outcomes were analysed using the intention-to-treat principle excluding dropouts as they had withdrawn their consent to use their data. Missing data were imputed using the last observations carried forward. Other outcomes were analysed using per protocol analysis. Stata version 14 was used for the analysis.

The Shapiro-Wilk test was used to determine the normality of data. As some baseline characteristics (e.g. age), efficacy and feasibility outcomes (e.g. time) were nonparametric, they were presented as median and interquartile range. Other baseline characteristics (e.g. medicine) and safety outcomes were presented as frequencies and percentages. The latter was also presented as odds ratios with 95% confidence intervals.

The Wilcoxon sum rank test was used to compare nonparametric variables, and *χ*^2^ test for categorical variables. Efficacy outcomes were measured repeatedly across time and analysed using generalised linear mixed models (GLMM), adjusting for their variability within and across participants and reducing the confounding effect of time. Statistical significance was set at *p* < 0.05. The trial ended when the last participant ended his follow-up.

## Results

### Baseline characteristics

Two hundred sixty patients were recruited and randomised. Of these, 205 patients (78.8%) were followed up to the day of discharge, with 10 patients (3.8%) lost to follow up at day 28 postdischarge. 2 patients (0.8%) could not comply to the study protocol. 7 patients (2.7%) dropped out during the study. 49 patients (18.8%) were hospitalised, of which 2 deaths occurred in the intervention group. However, the dropouts, hospitalisations or deaths were unrelated to any side effects of the intervention (Table 4).

Two fifty three patients were analysed excluding dropouts. Baseline characteristics were similar in both groups, except that the intervention group (*n* = 126) had more patients with a greater length of stay [Median (IQR): 27 (17.5, 38) versus 22 (13, 32); *p* = 0.040] and had Paracetamol as the initial deprescribing target [88.1% versus 73.2%, *p* = 0.009] (Table 1 & Supplementary Table).

**Table 1:** Baseline Characteristics

	Intervention (***N*** = 126)	Control (***N*** = 127)
**Demographics**		
Age, Median (IQR)	76 (70, 81)	75 (70, 80)
Female, N (%)	73 (58.7)	76 (59.8)
**Ethnicity**		
Chinese, N (%)	109 (86.5)	112 (88.2)
Malay, N (%)	8 (6.4)	7 (5.5)
Indian, N (%)	8 (6.4)	8 (6.3)
Eurasian, N (%)	1 (0.8)	0 (0.0)
**Discipline**		
Rehabilitation, N (%)	102 (81.0)	100 (78.7)
Subacute, N (%)	24 (19.1)	27 (21.3)
**Other parameters**		
AMT Score, Median (IQR)	10.0 (9, 10)	10.0 (9, 10)
Length of Stay in days, Median (IQR)^c^	27 (17.5, 38)^a^	22 (13, 32)^b^
**Baseline medicine parameters**		
Total Daily Dose (TDD), Median (IQR)	23 (18, 28)	23 (18, 29)
Total Number of Medicine (TNM), Median (IQR)	13 (11, 16)	13 (10, 17)
Total Daily Cost (TDC) in S$, Median (IQR)	5.94 (4.32, 9.08)	6.18 (3.98, 9.74)

^a^n = 124; ^b^n = 127; ^c^p = 0.040

Note: p is ≥0.05 for all characteristics except for length for stay

IQR: Interquartile range

Most medicine changes were small and consists of reduction of dosing frequency (e.g. reducing gabapentin 300 mg from thrice to twice daily).

### Primary outcome

There was a median change of − 19.62% (Interquartile Range [IQR]: − 34.38, 0.00%) in TDD upon discharge from baseline in the intervention group, compared to 0.00% (IQR: − 12.00, 6.82%) in the control group. (*p* < 0.001) (Table 2).

**Table 2:** Efficacy Outcomes

A. Percentage change from baseline for medicine parameters expressed in median (interquartile range)				
Outcome	Phases	Intervention (N = 126)	Control (N = 127)	***p*** value
**Total Daily Dose (TDD)**	Inpatient phase, day 14 postrecruitment	– -12.50 (−27.27, 0.00)	0.00 (−11.43, 6.67)	< 0.001
	Inpatient phase, day 28 postrecruitment	−14.91 (−32.00, 0.00)	0.00 (− 11.76, 7.14)	< 0.001
	Discharge day **(Primary Outcome)**	− 19.62 (− 34.38, 0.00)	0.00 (− 12.00, 6.82)	< 0.001
	Outpatient phase, day 28 postdischarge	− 22.54 (− 41.18, 0.00)	–7.69 (− 28.57, 0.00)	0.001
**Total Number of Medicine (TNM)**	Inpatient phase, day 14 postrecruitment	– 5.26 (− 16.67, 0.00)	0.00 (−9.09, 5.88)	0.008
	Inpatient phase, day 28 postrecruitment	0.00 (− 18.18, 5.56)	0.00 (−10.00, 5.88)	0.035
	Discharge day	– 5.56 (−20.00, 0.00)	0.00 (−11.76, 5.88)	0.035
	Outpatient phase, day 28 postdischarge	– 7.14 (−23.08, 0.00)	0.00(−16.67, 5.56)	0.203
**Total Daily Cost (TDC)**	Inpatient phase, day 14 postrecruitment	– 8.91 (−27.55, 0.00)	0.00 (−14.99, 3.57)	0.004
	Inpatient phase, day 28 postrecruitment	−10.66 (−35.86, 0.00)	0.00 (−15.83, 5.63)	0.002
	Discharge day	−14.74 (−38.22, 0.00)	0.00 (−23.90, 7.60)	0.001
	Outpatient phase, day 28 postdischarge	−17.31 (−47.07, 0.00)	– 7.61 (−37.63, 1.80)	0.116
**B: Analysis of the change of TDC/TNM/TDC across time using GLMM**				
		**Regression Coefficient (95% CI)**	**p value**	
**TDD**	Unadjusted group effects	−2.836 (−4.888, −0.785)	0.007	
	Adjusted* group effects	−3.113 (−5.153, −1.072)	0.003	
**TNM**	Unadjusted group effects	−0.830 (−1.875, 0.216)	0.120	
	Adjusted group effects	−0.994 (−2.046, 0.0587)	0.064	
**TDC**	Unadjusted group effects	−3.564 (−10.882, 3.754)	0.340	
	Adjusted group effects	−3.585 (−10.830, 3.661)	0.332	

Note: Percentage change from baseline is calculated for every individual participant before their collective median (IQR) is computed

*Adjusted for repeated measurements throughout the study

### Secondary outcomes

On day 28 postdischarge, the median change inTDD improved to − 22.54% (IQR: − 41.18, 0.00%) in the intervention group, compared to − 7.69% (IQR: − 28.57, 0.00%) in the control group (*p* = 0.001).

There was a change of − 5.56% in TNM upon discharge from baseline in the intervention group, compared to 0.00% in the control group (*p* = 0.035) and this improved to − 7.14% (intervention) and 0.00% (control) on day 28 postdischarge (*p* = 0.203).

There was a change of − 14.74% in TDC upon discharge from baseline in the intervention group, compared to 0.00% in the control group (p = 0.001) and this improved to − 17.31% (intervention) and − 7.61% (control) on day 28 postdischarge (*p* = 0.116).

On day 14 postrecruitment, there was an improvement in all efficacy outcomes from baseline for the intervention group (TDD: -12.50%, TNM: -5.26%; TDC: − 8.91%), compared to the control group (TDD: 0.00%, TNM: 0.00%; TDC: 0.00%) (*p* < 0.050). However, on day 28 postrecruitment, there was no change in TNM from baseline for both the intervention and control groups, unlike TDD (Intervention: − 14.91%; Control: 0.00%) and TDC (Intervention: − 10.66%; Control: 0.00%) (p < 0.050).”

Using GLMM, there was a consistent reduction of TDD across time in the intervention, compared to the control group (Coefficient: − 3.113 (− 5.153, − 1.072), *p* = 0.003) but this did not apply to TNM and TDC. This coefficient reflects the average gradient of change of the outcome variable with time.

For safety outcomes, more constipation (OR: 3.75, 95% CI:1.75–8.06, *p* < 0.001) (Table 3) and laxatives represcription (OR: 2.82, 95% CI:1.30–6.12, *p* = 0.009) occured in the intervention group (Table 4). However, other adverse events were not significantly higher in the intervention group. Hospitalisation rates are similar in both groups (Intervention: 18.3%; Control: 20.4%; OR: 0.87, 95% CI: 0.46–1.62, *p* = 0.655) (Table 3) and were unrelated to deprescribing. The 2 deaths in the intervention group were unrelated to deprescribing and were due to terminal malignancy and pneumonia.

**Table 3:** Safety Outcomes

A. Medicine associated with symptom recurrence after deprescribing						
	**Intervention Group**		**Control Group**		**Odds Ratio** **(95% CI)**	**p value**
	**Number of patients with target medicine initially deprescribed** **N**	**Number of patients with symptom recurrence** **n (%)**	**Number of patients with target medicine initially deprescribed** **N**	**Number of patients** **with symptom recurrence** **n (%)**		
Painkillers	102	24 (23.5)	76	11 (14.5)	1.80 (0.83, 3.99)	0.140
Laxatives	80	51 (63.8)	47	15 (31.9)	3.75 (1.75, 8.06)	< 0.001
Antiemetics	37	5 (13.5)	31	3 (9.7)	1.46 (0.32, 6.66)	0.630
Gastroprotectives	40	8 (20.0)	32	7 (21.9)	0.89 (0.29, 2.80)	0.850
Steroid Creams	10	4 (40.0)	13	4 (30.8)	1.50 (0.27, 8.45)	0.650
Vitamin B based supplements	19	2 (10.5)	4	1 (25.0)	0.35 (0.02, 5.23)	0.450
Glucosamine	6	0 (0.0)	0	0 (0.0)	N.A.	N.A.
Multivitamins	1	0 (0.0)	0	0 (0.0)	N.A.	N.A.
Diuretics	4	0 (0.0)	4	2 (50.0)	N.A.	N.A.
Benzodiazepines	4	2 (50.0)	2	1 (50.0)	1.00 (0.03, 29.81)	1.000
Antihistamines (for insomnia)	3	0 (0.0)	3	1 (33.3)	N.A.	N.A.
Antihistamines (for itch)	5	2 (40.0)	4	1 (25.0)	2.00 (0.11, 35.81)	0.640
Opioids (for Cough)	18	2 (11.1)	21	3 (14.3)	0.75 (0.11, 5.07)	0.770
Opioids (for diarrhoea)	1	1 (100.0)	3	2 (66.7)	N.A.	N.A.
**B. Medicine which are restarted or substituted after deprescribing**						
	**Intervention**		**Control**		**Odds Ratio** **(95% CI)**	**p** **value**
	**Number of patients with target medicine initially deprescribed** **n**	**Number of patients with medication restarted/ substituted** **n (%)**	**Number of patients with target medicine initially deprescribed** **n**	**Number of patients with medication restarted/ substituted** **n (%)**		
Painkillers	102	67 (65.7)	77	46 (59.7)	1.29 (0.70, 2.38)	0.410
Laxatives	84	39 (46.4)	51	12 (23.5)	2.82 (1.30, 6.12)	0.009
Antiemetics	37	5 (13.5)	31	6 (19.4)	0.65 (0.18, 2.38)	0.520
Gastroprotectives	44	7 (15.9)	34	9 (26.5)	0.53 (0.17, 1.60)	0.260
Steroid Creams	10	3 (30.0)	13	6 (46.2)	0.50 (0.09, 2.84)	0.430
Vitamin B based supplements	18	3 (16.7)	4	0 (0.0)	N.A.	N.A.
Glucosamine	7	2 (28.6)	0	0 (0.0)	N.A.	N.A.
Multivitamins	1	0 (0.0)	0	0 (0.0)	N.A.	N.A.
Diuretics	5	0 (0.0)	5	1 (20.0)	N.A.	N.A.
Benzodiazepines	5	1 (20.0)	2	0 (0.0)	N.A.	N.A.
Antihistamines (for insomnia)	4	2 (50.0)	3	2 (66.7)	0.50 (0.02, 11.09)	0.660
Antihistamines (for itch)	6	2 (33.3)	5	0 (0.0)	N.A.	N.A.
Opioids (for cough)	19	3 (15.8)	23	7 (30.4)	0.43 (0.09, 1.96)	0.270
Opioids (for diarrhoea)	2	1 (50.0)	3	2 (66.7)	0.50 (0.01, 19.56)	0.710
**C: Hospitalisation and deaths**						
	**Intervention (N = 126)**	**Control (N = 127)**	**Odds Ratio** **(95% CI)**	**p** **value**		
Hospitalisations, n (%)	23 (18.3)	26 (20.4)	0.87 (0.46, 1.62)	0.655		
Deaths, n (%)	2 (1.6)	0 (0)	N.A.	0.247

**Table 4:** Reasons for hospitalisations, deaths and dropouts

	Intervention	Control
Hospitalisations	***N*** **= 23** Elective cholangiopancreatogram (n = 1) Fluid overload with pneumonia (*n* = 1) Pleural effusion (*n* = 1) Sepsis (*n* = 5) Haemoptysis for workup (*n* = 1) Worsening neuropathy (*n* = 1) Suspected stroke (*n* = 1) Rectal bleeding for workup (n = 1) Suspected fracture (n = 1) Suspected septic arthritis (n = 2) Suspected myocardial infarction (n = 1) Worsening wound infection (n = 1) Altered mental state for workup (n = 1) Worsening gangrene (n = 1) Suspected deep vein thrombosis (n = 1) Fluid overload (n = 1) Pneumonia (n = 1) * Lung Cancer (n = 1) *	***N*** **= 26** Elective knee replacement (n = 1) Elective nephrectomy (n = 1) Fast atrial fibrillation (n = 1) Suspected deep vein thrombosis (n = 1) Worsening renal impairment (n = 1) Removal of central venous catheter (n = 1) Worsening anaemia (n = 3) Sepsis (n = 2) Fluid overload (n = 1) Suspected implant infection (n = 1) Suspected myocardial (*n* = 3) Worsening numbness (n = 1) Pneumonia with seizures (n = 1) Severe hyponatremia (n = 1) Fluid overload, pneumonia & fast AF (n = 1) Worsening ascites(n = 1) Incarcerated hernia (n = 1) Worsening fracture (n = 1) Hematemesis (n = 1) Finger abscess (n = 1) Intestinal obstruction (n = 1)
Deaths	**N = 2** Pneumonia (n = 1) Lung Cancer (n = 1)	
Dropouts	***N*** **= 4** Patient felt study was not helpful to him (n = 1) Patient prefers to continue current medicine (n = 3)	**N = 3** Patients felt study was not helpful to them (n = 2) Patient prefers to continue usual medicine (n = 1)

*demised

For feasibility outcomes, the median rounding time was 7.09 min per patient (IQR: 5.21–9.19 min). Common challenges include the inconvenience of assembling the team for rounding and spending time to address the concerns of patients regarding deprescribing.

We also gathered feedback from patients and ward doctors. The patients were generally receptive to the team’s recommendation, although many were initially hesitant to deprescribing as they had been taking the targets for sometime and feared side effects. Patient education, gradual tapering of doses and the assurance that medicine would be promptly re-prescribed for symptom recurrence helped to alley these fears. The ward doctors also welcomed the additional help rendered by the team. Although there were few [5] disagreements on the team’s recommendations without any safety implications, the team respected the ward doctors’ opinions and that helped to reassure them.

## Discussion

We demonstrated that deprescribing rounds resulted in a 19.62% reduction in TDD and 14.74% reduction in TDC upon discharge.

Our study showed comparable hospitalisation rates in both groups. This is similar to Edey’s paper which explored the impact of pharmacist-led deprescribing rounds in a Canadian hospital [20]. Moreover, our study was able to demonstrate the potential of deprescribing rounds in reducing medicine cost. This was similarly shown in William’s study, which involved a medicine review by a pharmacist before the recommendations are adjusted by a multidisciplinary team, endorsed and recommended to clinic patients by the primary physician. However, its cost savings may differ, depending on the selling price of the wholesale medicine, predetermined deprescribing targets and the patients’ perceptions towards deprescribing [24].

The reduction in TNM was modest (5%) upon discharge with our intervention. This is incongruent with the 15% decrease in drug use in Roberts’ study whose intervention involved developing interprofessional relationships, educating nurses in medication matters and individualising medication review and deprescribing for nursing home residents [23]. One reason is that the intervention resulted in a greater reduction of dosing frequency rather than the absolute cessation of medicine. Moreover, our study reported more constipation (OR: 3.75) compared to Ee’s study (calculated OR: 0.899) [19]. The latter’s intervention was a pharmacist-led deprescribing review of symptomatic medicine in a rehabilitative hospital. Possible reasons for more constipation reported in our study include the small sample size, utilisation of a single point pharmacist-led intervention and cessation of data collection upon inpatient discharge for the latter’s study.

We observed some interesting findings. First, there was a sustained reduction in TDD and TDC in both groups after discharge, although the latter was statistically insignificant. One reason could be that the requirements and hence dosing frequencies of symptomatic medicine had reduced as the patients continued to recover postdischarge. This is supported by the fact that the change in TNM in both groups remained similar upon discharge and postdischarge. Secondly, there were no reduction in TNM in both groups on day 28 postrecruitment. These effects were similar in the later period of deprescribing in 4 local nursing homes based on Kua’s study [30]. One explanation could be that the remaining inpatients might have more treatment, thus nullifying the effects of deprescribing. Lastly, we noticed the median change in all efficacy endpoints in the control group was 0.00% across all inpatient time points. One explanation is that deprescribing practices by many ward teams in the control group was very varied and may not be regular or deliberate.

The potential impact of our results is a fewfold, though this may apply to other methods of deprescribing. Firstly, the reduction of TDD translates to reduced pill burden, which could encourage medicine adherence and reduce medication error. Next, there would be healthcare savings at the individual, institutional and national levels, whilst preserving patient safety. Thus deprescribing rounds could help to address the challenges of rising healthcare utilisation faced by ageing countries such as Singapore [17].

The strengths of our study were described in the measures taken to mitigate selection, detection and reporting biases. Moreover, its open label nature and involvement of clinicians and patients in deprescribing ensured safety and accountability.

Several limitations exist. First, the outcomes were not adjusted for all baseline differences, including the percentage of patients having Paracetamol as the initial target. This may overestimate the efficacy and side effects of the intervention. However, we conducted further analysis using GLMM which showed similar reduction in TDD across time to the primary analyses. Secondly, the overall rate of study discontinuation and loss to follow up was greater than 20%. However, for most outcomes, we applied the intention-to-treat analysis, hence maintaining prognostic balance in both groups. Thirdly, the followup duration was one month, whereas most studies lasted for a weighted mean of 15.5 months [28]. Thus, the sustained effects of our intervention remain unknown.

Next, investigators form part of the multidisciplinary deprescribing team and this study is conducted in a Singapore rehabilitation hospital setting, thus limiting the generalisability and adaptability of the intervention to other practice settings or other Asian countries. This study did not address the other costs involved in deprescribing (e.g. manpower cost, processing of re-prescriptions, cost incurred in addressing side effects) and thus could not advice on its cost-effectiveness. Restricting deprescribing targets to a standard list may compromise the patient-centredness approach as deprescribing medicine outside the list may potentially be more important to the patient and more congruent with the goals of care. Also, scaling up dedicated deprescribing rounds locally may be initially challenging given manpower constraints and differing work schedules of the multidisciplinary team, though one possible solution is to deliberately integrate such deprescribing into usual rounds. Moreover, the open label nature of our trial introduced bias as clinicians in both groups may compete in or omit deprescribing and patients may not accurately report their symptoms after knowing their allocation. Nonetheless, we desired partnership of the ward team and patients as they need to know the deprescribing intervention. Hence, the ward team and patients were not blinded.

## Conclusion

Deprescribing rounds can safely reduce TDD of medicine upon discharge compared to usual care in a Singaporean rehabilitation hospital. Further studies are required to evaluate its use in other practice settings or other countries.

## Supplementary Information

**Additional file 1.** Supplementary Table: Medical Diagnoses & Initial List of Medicine

## Data Availability

The datasets used and analysed during this study are available from the corresponding author on reasonable request.

## Abbreviations

AMT: Abbreviated Mental Test
CI: Confidence Interval
GLMM: Generalised Linear Mixed Models
IQR: Interquartile Range
OR: Odds Ratio
TDC: Total Daily Cost
TDD: Total Daily Dose
TNM: Total Number of Medicine
