# Supplementary material for: Feasibility & Efficacy of Deprescribing rounds in a Singapore rehabilitative hospital- a randomised controlled trial
Source: BMC Geriatr. 2021 Oct 21;21:584. doi: 10.1186/s12877-021-02507-0 (PMC8529728; doi:10.1186/s12877-021-02507-0)
Supplement: Supplementary file 1 — Additional file 1. Supplementary Table: Medical Diagnoses & Initial List of Medicine [file 12877_2021_2507_MOESM1_ESM.doc]

Supplementary Index

| **Supplementary Table: Medical Diagnoses and List of medicine** | |  |
| --- | --- | --- |
| 1. **Medical Diagnoses** |  |  |
|  | **Intervention, n (%)** | **Control, n (%)** |
| ***Respiratory diseases*** |  |  |
| Chronic Obstructive Lung Disease | 5 (4.0) | 2 (1.6) |
| Bronchial Asthma | 2 (1.6) | 8 (6.3) |
| **Cardiovascular Risk Factors** | | |
| Diabetes Mellitus | 41 (32.5) | 40 (31.5) |
| Hypertension | 94 (74.6) | 99 (78.0) |
| Hyperlipidaemia | 77 (61.1) | 83 (65.4) |
| **Cardiovascular diseases** | | |
| Atrial Fibrillation | 18 (14.3) | 10 (7.9) |
| Congestive Heart Failure | 7 (5.6) | 14 (11.0) |
| Stroke | 26 (20.6) | 23 (18.1) |
| Ischemic Heart Disease | 26 (20.6) | 32 (25.2) |
| Peripheral Arterial Disease | 8 (6.4) | 5 (3.9) |
| **Rheumatologic and orthopaedic diseases** | | |
| Gout | 8 (6.4) | 10 (7.9) |
| Osteoporosis | 13 (10.3) | 18 (14.2) |
| Osteoarthritis | 42 (33.3) | 46 (36.2) |
| Rheumatoid Arthritis | 2 (1.6) | 4 (3.2) |
| Cervical or Lumbar Spondylosis | 17 (13.5) | 29 (22.8) |
| Fractures | 51 (40.5) | 37 (29.1) |
| **Neurological diseases** | | |
| Cognitive Impairment | 14 (11.1) | 8 (6.3) |
| Parkinsonism | 7 (5.6) | 5 (3.9) |
| Fits | 1 (0.8) | 0 (0.0) |
| Cervical Myelopathy | 3 (2.4) | 5 (3.9) |
| Peripheral Nerve Disorders | 3 (2.4) | 6 (4.7) |
| **Psychiatric Diseases** | | |
| Anxiety | 0 (0.0) | 4 (3.2) |
| Depression | 4 (3.2) | 6 (4.7) |
| Bipolar Disorder | 1 (0.8) | 0 (0.0) |
| Schizophrenia | 1 (0.8) | 0 (0.0) |
| **Gastrointestinal Diseases** | | |
| Gastritis | 12 (9.5) | 16 (12.6) |
| Gastroesophageal Reflux | 3 (2.4) | 3 (2.4) |
| Gallstone Disease | 7 (5.6) | 4 (3.2) |
| Upper Gastrointestinal Bleed | 4 (3.2) | 2 (1.6) |
| **Oncological diseases** | | |
| Malignancies | 23 (18.3) | 17 (13.4) |
| Premalignancies | 1 (0.8) | 3 (2.4) |
| **Other diseases** | | |
| Benign Prostatic Hypertrophy | 10 (7.9) | 8 (6.3) |
| Dermatitis | 9 (7.1) | 9 (7.1) |
| Chronic Venous Insufficiency | 3 (2.4) | 3 (2.4) |
| Chronic Kidney Disease | 22 (17.5) | 23 (18.1) |
| Chronic Liver Disease | 1 (0.8) | 2 (1.6) |

Note: p is ≥0.05 for all diagnoses

|  |  |  |
| --- | --- | --- |
| **B: Initial List of Medicine** |  |  |
|  | **Intervention, n (%)** | **Control, n (%)** |
| **Supplements** | | |
| Glucosamine | 10 (7.9) | 19 (15.0) |
| Multivitamin | 32 (25.4) | 44 (34.7) |
| Vitamin B predominant supplements | 4 (3.2) | 3 (2.4) |
| **Painkillers** | | |
| Paracetamol^a^ | 111 (88.1) | 93 (73.2) |
| Paracetamol/ Orphenadrine (Anarex®) | 3 (2.4) | 8 (6.3) |
| Eperisone | 1 (0.8) | 0 (0.0) |
| Baclofen | 0 (0.0) | 2 (1.6) |
| Nonselective NSAIDs | 2 (1.6) | 2 (1.6) |
| COX2 Inhibitors | 14 (11.1) | 14 (11.0) |
| Topical Analgesia | 43 (34.1) | 45 (35.4) |
| Tramadol | 52 (41.3) | 51 (40.2) |
| Codeine | 1 (0.8) | 1 (0.8) |
| Morphine | 6 (4.8) | 4 (3.2) |
| Fentanyl Patch | 0 (0.0) | 1 (0.8) |
| GABA Inhibitors | 29 (23.0) | 39 (30.7) |
| Tricyclic Antidepressants | 0 (0.0) | 1 (0.8) |
| **Topical Steroids** | | |
| Steroid Creams | 8 (6.4) | 9 (7.1) |
| **Gastroprotectives** | | |
| Antacids | 11 (8.7) | 8 (6.3) |
| H_2_ Blockers | 12 (9.5) | 21 (16.5) |
| Proton Pump Inhibitors | 69 (54.8) | 72 (56.7) |
| **Laxatives** | | |
| Lactulose | 101 (80.2) | 96 (75.6) |
| Sennosides | 79 (62.7) | 72 (56.7) |
| Bisacodyl | 42 (33.3) | 55 (43.3) |
| Macrogol | 2 (1.6) | 4 (3.2) |
| Ispaghula (Fybogel®) | 0 (0.0) | 1 (0.8) |
| **Anti-emetics** | | |
| Metoclopramide | 34 (27.0) | 36 (28.4) |
| Domperidone | 1 (0.8) | 0 (0.0) |
| Prochlorperazine | 1 (0.8) | 0 (0.0) |
| Cinnarizine | 1 (0.8) | 1 (0.8) |
| Flunarizine | 0 (0.0) | 1 (0.8) |
| Haloperidol | 0 (0.0) | 2 (1.6) |
| **Beer's List: Diuretics** | | |
| Furosemide | 11 (8.7) | 14 (11.0) |
| **Beer's List: Nonanalgesic opioids** | | |
| Antitussives | 8 (6.4) | 11 (8.7) |
| Antidiarrhoeals | 1 (0.8) | 2 (1.6) |
| **Beer's List: Benzodiazepines** | | |
| Benzodiazepines | 8 (6.4) | 5 (3.9) |
| **Beer's List: Alpha Blockers** | | |
| Prazosin | 2 (1.6) | 0 (0.0) |
| Terazosin | 1 (0.8) | 0 (0.0) |
| **Beer's List: Antihistamines** | | |
| Hydroxyzine | 6 (4.8) | 2 (1.6) |
| Chlorpheniramine | 1 (0.8) | 2 (1.6) |
| ^a^ P=0.009  Note: p is ≥0.05 for all medicine except Paracetamol.  NSAIDs: Nonsteroidal Antiinflammatory Agents  COX2: Cyclooxygenase 2  GABA: Gama Amino Butyric Acid  H_2_: Histamine type 2 receptor | | |
